# A data set for electric power consumption forecasting based on socio-demographic features: Data from an area of southern Colombia

**DOI:** 10.1016/j.dib.2020.105246

**Published:** 2020-02-06

**Authors:** Jorge Parraga-Alava, Jorge Dario Moncayo-Nacaza, Javier Revelo-Fuelagán, Paul D. Rosero-Montalvo, Andrés Anaya-Isaza, Diego Hernán Peluffo-Ordóñez

**Affiliations:** a Facultad de Ciencias Informáticas, Universidad Técnica de Manabí, Portoviejo, Ecuador; b Universidad de Buenos Aires, Buenos Aires, Argentina; c Universidad de Nariño, Pasto, Colombia; d Universidad Técnica del Norte, Ibarra, Ecuador; e Instituto Tecnológico Superior 17 de Julio, Ibarra, Ecuador; f INDIGO Research, Bogotá, Colombia; g Pontificia Universidad Javeriana, Bogotá, Colombia; h Corporación Universitaria Autónoma de Nariño, Pasto, Colombia; i Universidad Yachay Tech, Urcuquí, Ecuador

**Keywords:** Electric power consumption, Forecasting, Machine learning, Socio-demographic data, Smart grid

## Abstract

In this article, we introduce a data set concerning electric-power consumption-related features registered in seven main municipalities of Nariño, Colombia, from December 2010 to May 2016. The data set consists of 4427 socio-demographic characteristics, and 7 power-consumption-referred measured values. Data were fully collected by the company Centrales Eléctricas de Nariño (CEDENAR) according to the client consumption records. Power consumption data collection was carried following a manual procedure wherein company workers are in charge of manually registering the readings (measured in kWh) reported by the electric energy meters installed at each housing/building. Released data set is aimed at providing researchers a suitable input for designing and assessing the performance of forecasting, modelling, simulation and optimization approaches applied to electric power consumption prediction and characterization problems. The data set, so-named in shorthand PCSTCOL, is freely and publicly available at https://doi.org/10.17632/xbt7scz5ny.3.

**Table:** Specifications Table

Subject	Energy.
Specific subject area	Power consumption, forecasting methods.
Type of data	Table.
How data were acquired	Power consumptions readings (PCR) are produced by electric energy meters installed at each client's housing/building by the company Centrales Eléctricas de Nariño S.A. E.S.P. – CEDENAR. As conventionally done in Colombia, such PRCs are collected by of means a manual inspection of meters records by CEDENAR's workers. Then, information is listed and registered by matching PRCs with corresponding client's socio-demographic information, as it is required for CEDENAR internal reports.
Data format	Raw and Processed CSV format.
Parameters for data collection	All available and provided data from CEDENAR internal reports were structured to create the data set. The only exception occurs in case that a client has no any electric energy meter installed. In this regard, as a general and widely accepted rule, the power consumption was estimated as the average installed electric load regarding the used/connected appliances -indeed this is how CEDENAR proceeds.
Description of data collection	Power consumption is obtained by manually registering the amount of kWh extracted from the monthly readings of the electric energy meters. Socio-demographic data were collected from the records available in CEDENAR internal reports (databases). Herein, a categorization of the clients' socioeconomic level (known as stratum in Colombia) is considered, just as suggested by the Colombian Law Book 732 of 2002 [1], namely: 0 = Low-low, 1 = Low, 2 = Medium-Low, 3 = Medium, 4 = Medium-High, 5 = high. Note that because the municipalities considered in the study do not have a high socio-economic level, the stratum data only have values between 0 and 3.
Data source location	Centrales Eléctricas de Nariño S.A. E.S.P. - CEDENAR, Nariño, Colombia. Latitude 1.2136101 and Longitude −77.2811127
Data accessibility	Repository name: PCSTCol - Power Consumption Data from an area of southern Colombia Data identification number: https://doi.org/10.17632/xbt7scz5ny.3 Direct URL to data: https://data.mendeley.com/datasets/xbt7scz5ny/2

**Table:** 

**Value of the Data** • Provided data enables both electric balance research and client behavior forecasting allowing for in-context, instantaneous strategies aimed at energy saving, and energy efficiency increasing. • As a matter of fact, the information about energy consumption patterns, and sociodemographic factors is crucial for regional planning, and effective socio-economic development. • Data can be used to forecast the power consumption using different forecasting models. • Given the mixed nature of the provided data set, it results interesting and appealing for power consumption applications involving training, testing and validation of both classification and clustering algorithms. • Data is suitable for modelling and simulation of different types of microgrids models. • Data is useful for benchmarking of algorithms for power consumption optimization, especially for multi-objective and mixed (quantitative and qualitative together) data formulations.

## 1 Data description

**Fig. 1:**
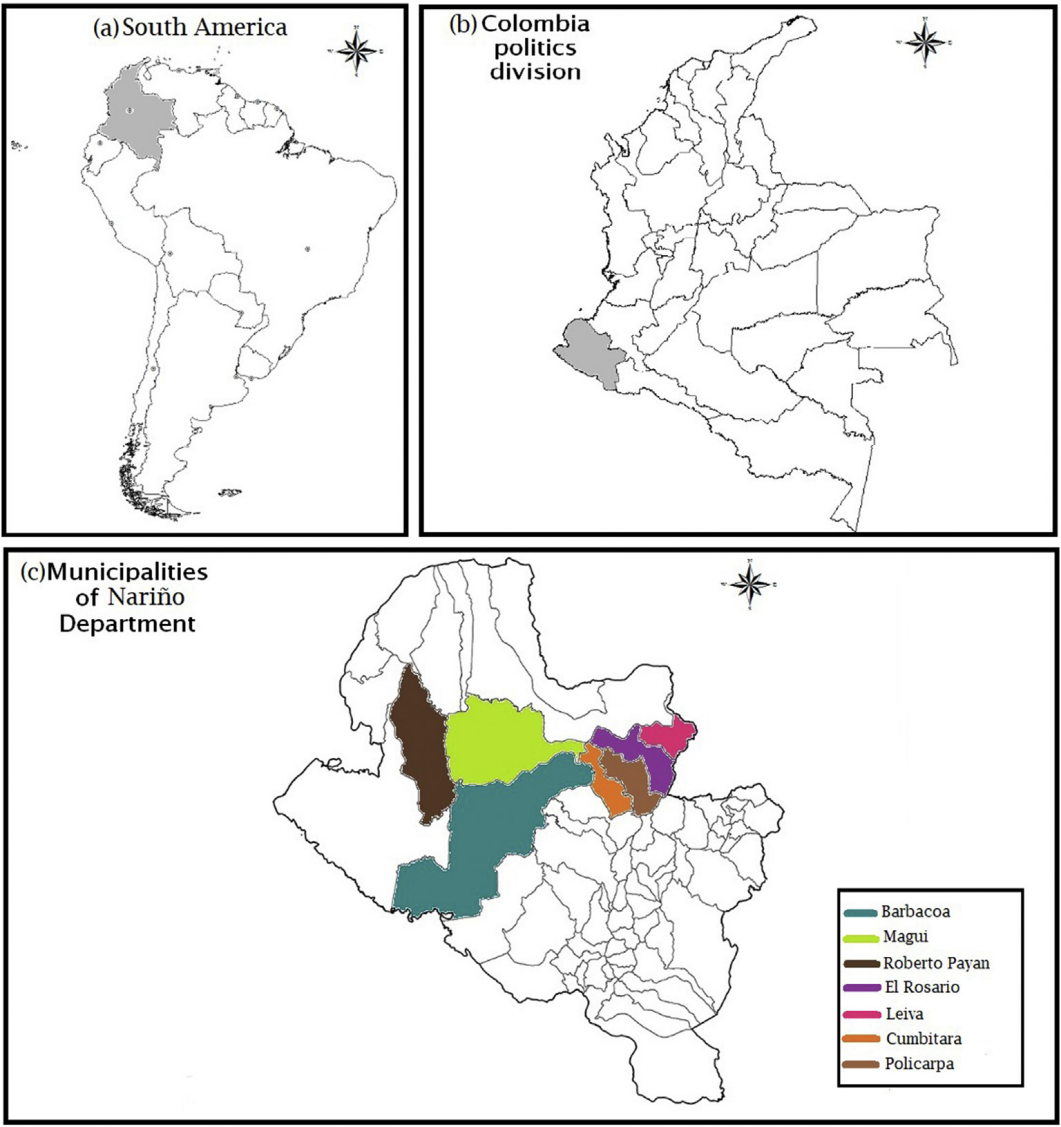
Geographical location of the study area regarding continental, country and regional maps: a) South America consisting of 12 countries, b) Colombia consisting of 32 provinces or departments, c) Municipalities of Department of Nariño where socio-demographic features and power consumption data were obtained from.

The data provides information about power consumption including area, date, municipality, use, stratum, and consumption of clients in the Departamento de Nariño, Colombia. The data set provides variables that can be used to train and validate the performance of machine learning algorithms used in regression and classification as well as modelling, simulation and optimization problems related to power consumption. The data set contains numerical and categorical data collected from December 2010 to May 2016 in seven municipalities available in the study area, as depicted in Fig. 1. In total, 4427 instances of power consumption by clients are included in the data set.

**Fig. 2:**
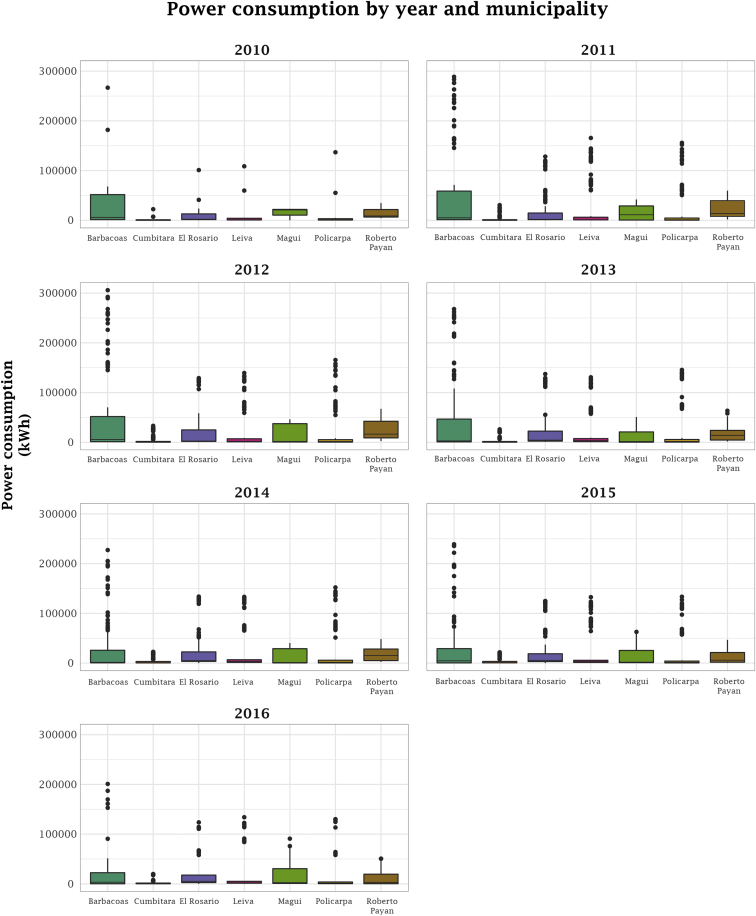
Range of power consumption values by municipality and year.

**Fig. 3:**
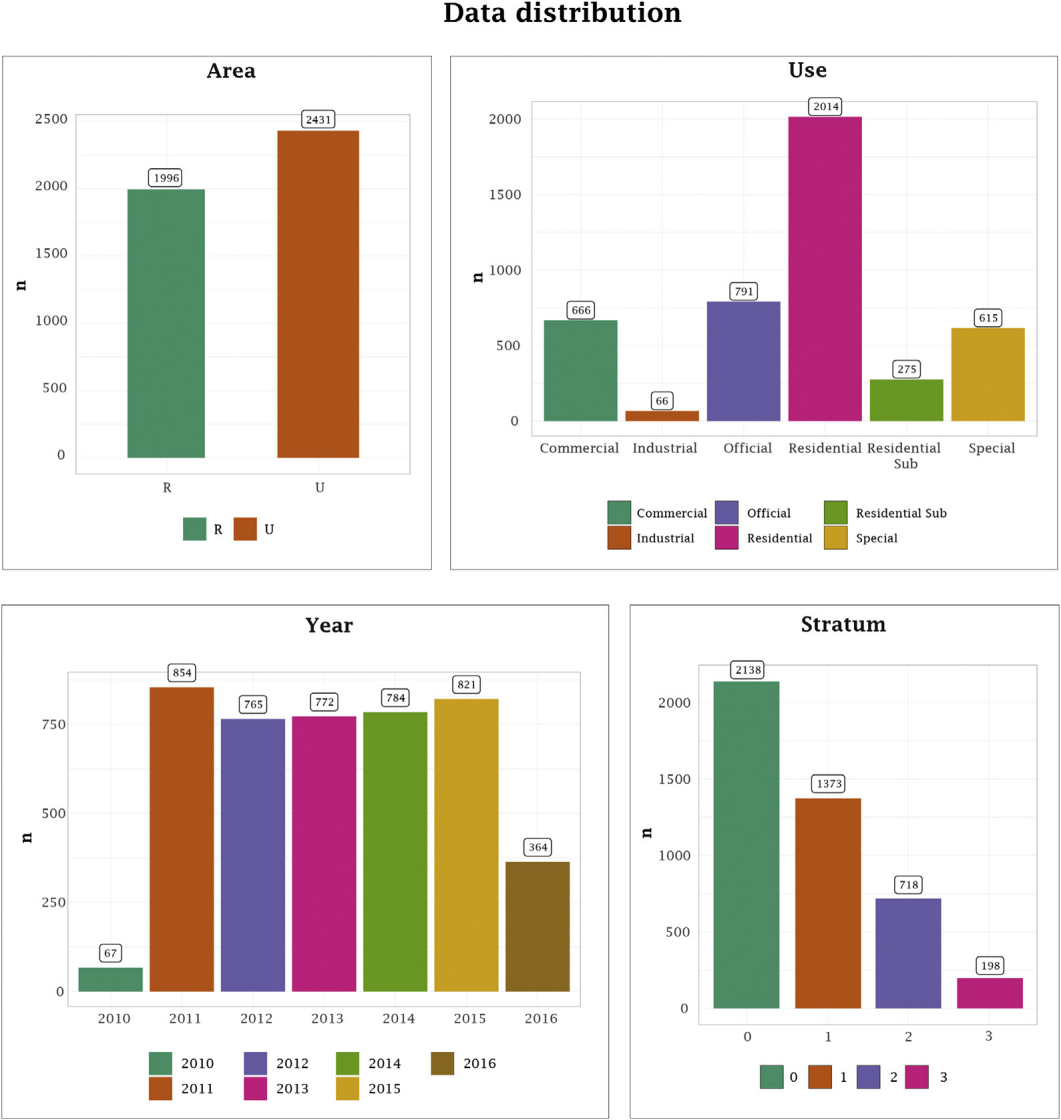
Data distribution of some features available in the data set.

Fig. 1 shows a visual representation of the geographical location of the municipalities of southern Colombia where the data are obtained from. Specifically, this area of study is formed by the municipalities: Barbacoas, Cumbitara, El Rosario, Leiva, Magui, Policarpa, and Roberto Payan. Next, in the Experimental Design, Materials, and Methods section, we describe in detail the socio-demographic and power consumption values that finally set the collection of features available in our data set, which are explained in Table 1. To give a general view about how the data matrix is structured, Table 2 presents few instances as an example. Also, in order to enable data set users to relate the structured information with additional geographic data, the information about geographic location, population, and selected instances for each municipality is gathered in Table 3. In addition, Fig. 2 and Fig. 3 provide a graphical depicting of the yearly power consumption ranges per municipality and a data distribution chart of some features available in the data set, respectively. Finally, Table 4 summarizes the values ranging, and type of the data. Concretely, the released files for the so-named PCSTCOL data set are as follows: raw data (db_raw.xls), raw data with zero consumption cases (db_raw_final.xlsx), pre-processed data (db_power_consumption.csv), pre-processed data with zero consumption cases (db_power_consumption_zeros.csv), feature description (db_power_consumption_details.txt) and the R script for data preprocessing (preprocessing.R).

**Table 1:** Description of features available in data set.

Number	Feature	Description
1	Code	A unique identifier code for clients, which has no any legal or practical use. It has been created by this data release authors for data set structuring purposes.
2	Area	U: Urban area of the municipality. R: Rural area of the municipality.
3	Date	It corresponds to date when measure was registered. In format yyyy-dd-mm
4	Municipality	It can be one of the following municipalities: Barbacoas, Cumbitara, El Rosario, Leiva, Magui, Policarpa, Roberto Payan.
5	Use	Type of client. It can be: Residential, Residential Sub, Industrial, Official, Commercial, Special.
6	Stratum	It represents the social strata, they range from 0 to 3, with 0 being the lowest. It corresponds to: 0 = Low-low, 1 = Low, 2 = Medium-Low, 3 = Medium
7	Consumption	Power consumption in kWh. It is the predictor variable.

**Table 2:** Example of instances available in the data set.

Code	Area	Date	Municipality	Use	Stratum	Consumption
8UBARE0	U	31-01-2016	BARBACOAS	Special	0	75.608
C0UCUMR3	U	28-02-2011	CUMBITARA	Residential	3	75.672
C0RPOLR1	R	30-11-2014	POLICARPA	Residential	1	77.516
8UBARE0	U	29-02-2016	BARBACOAS	Special	0	77.936
C0UCUMR3	U	31-08-2011	CUMBITARA	Residential	3	78.370
C0RCUMR1	R	31-12-2010	CUMBITARA	Residential	1	78.674
C2UCUMR1	U	31-01-2013	CUMBITARA	Residential	1	79.379
C1RCUME0	R	31-08-2012	CUMBITARA	Special	0	79.570
C0RCUMR1	R	28-02-2013	CUMBITARA	Residential	1	79.835
C2RCUMO0	R	31-12-2010	CUMBITARA	Official	0	83.622
C0UCUMR3	U	31-01-2015	CUMBITARA	Residential	3	84.220
C1UPOLR1	U	31-10-2015	POLICARPA	Residential	1	84.303
C1RPOLO0	R	30-04-2016	POLICARPA	Official	0	84.708
C0UCUMC0	U	31-12-2012	CUMBITARA	Commercial	0	85.445

**Table 3:** Location, entire population and selected instances for each municipality.

Municipality	Latitude	Longitude	Population	Instances	Total in data set
Barbacoas	1.67262 (1° 40′ 21″ N)	−78.1393 (78° 8′ 21″ W)	30.256	782	17.66%
Cumbitara	1.7644 (1° 45′ 52″ N)	−78.1817 (78° 10′ 54″ W)	13.831	833	18.82%
El Rosario	1.69632 (1° 41′ 47″ N)	−78.2443 (78° 14′ 39″ W)	17.286	625	14.12%
Leiva	1.74192 (1° 44′ 31″ N)	−77.3352 (77° 20′ 7″ W)	11.204	659	14.89%
Magui	1.933 (1° 55′ 59″ N)	−77.3 (77° 18′ 0″ W)	11.825	345	7.79%
Policarpa	1.6548 (1° 39′ 17″ N)	−77.5842 (77° 35′ 3″ W)	6.142	866	19.56%
Roberto Payan	1.6279 (1° 37′ 40″ N)	−77.4589 (77° 27′ 32″ W)	9.798	317	7.16%

**Table 4:** Description of data set features.

Feature	Data type	Values range
Code	Character	Mixed character string
Area	Categorical	[R, U]
Date	Date	[2010-12-31 to 2016-05-31]
Municipality	Categorical	[Barbacoas, Cumbitara, El Rosario, Leiva, Magui, Policarpa, Roberto Payan]
Use	Categorical	[Residential, Residential Sub, Industrial, Official, Commercial, Special]
Stratum	Categorical	[0,1,2,3]
Consumption	Numerical	[1.009–305687.4]

## 2 Experimental Design, materials, and methods

The power consumption and socio-demographic features were acquired from the electric energy meter installed in each house/building of clients by Centrales Eléctricas de Nariño S.A. E.S.P. - CEDENAR. The data were collected from seven municipalities of Nariño-Colombia, namely: Barbacoas, Cumbitara, El Rosario, Leiva, Magui, Policarpa, and Roberto Payan, during the time period ranged from December 2010 to May 2016. In the study area, clients of both urban and rural zone were considered. Over the original data provided by CEDENAR, we carried out a pre-processing procedure where financial features as invoiced value, outstanding balance and public lighting rates were eliminated, which finally yielded the total of seven features detailed in Table 1.

Table 1. shows the descriptions of the socio-demographic features as well as the power consumptions values for each client of CEDENAR. At this point, it is worth noticing that features “Area”, “Municipality” and “Use” are categorical and characters, but they might easily be converted to discrete values. “Stratum” implies a socioeconomic classification according to the residential properties where there is some consumption of a public service. Stratification is used to differentiate costs for public services, which allows for allocating subsidies and collecting contributions by area. In this study, the level is ranged between 0 and 3 corresponding to Low-low, Low, Medium-Low, Medium, respectively, because the considered clients are on those levels only, being as a matter of fact the most representative population from the study area.

Broadly, different energy meters are in use throughout the electricity distribution system of the Department of Nariño.

Mostly, electronic active energy meters, being class 1.0 and reactive class 2.0 type, working at 120/208 v, and 1 or 5 A. There are also few multifunctional energy meters. To this work interests, there is no need for any distinction among the meters since their provided consumption readings are the same with no alterations regarding the either location or stratum.

Nonetheless, in order to a measurement of electrical consumption to be carried out, CEDENAR must guarantee the following criteria:

• The measuring elements are specified, installed, operated and maintained in accordance with the provisions of the official guideline (CREG 038 DEL 2014).
• The elements of the measurement system must comply with the requirements of accuracy, calibration and safety (physical and computer) established in the official guideline (CREG 038 DEL 2014).
• The workers involved in the information collection task must be properly trained to do so following recommendations by the Wholesale Energy Market (WEM).

Regulations aforementioned are stated in CEDENAR guidelines.^2^

Once the number of features was reduced to obtain socio-demographic features only, we performed a filtering procedure where instances with missing or inconsistent data were deleted. After pre-processing and filtering procedures about 6000 rows were eliminated. Most of these cases correspond to erroneous consumption over the years. For a practical use, zero values of power consumption were considered as erroneous and therefore removed. The resulting data set version at this stage is the one recommended in this paper for a direct use. The raw data also includes negative values. On this regard, the negative value of some power records means that an additional adjustment was carried out since visual inspection of power meters was either unfeasible or -anyhow- wrongly performed. This aspect is widely known and considered by South America protocols to manual collection of data.^2^ Alternatively, this data set version is also available so that the data-set's expert users can deal with the negative values adjustment, which entails the use of more challenging data transformation techniques.

Such an alternative version of the data set -conserving the same features-is described in Table 1 but with no applying any filtering procedure. As a consequence, the alternative data set version contains 10109 instances because it includes the power consumption with negative values. Notwithstanding, the main data set here-presented for power consumption forecasting purposes contains 4427 instances corresponding to clients of CEDENAR Company in seven municipalities in Nariño, Colombia.

As summary, in addition to the pre-processed version (undergone the whole filtering/pre-processing procedure and with including no negative values), we have kept the alternative well as raw data. A view of some instances from the data set is shown in Table 2. Further statements and final structure of the database are set accordingly the following notations:

• Code: It has been created by this data release authors for data set structuring purposes.
• Urban area (U): Area of the municipality wherein there is a greater population density and land extension, and provision of all types of infrastructure.
• Rural area (R): Zone of the municipality wherein the population is expansive, at different scales, to the territory of a region or a town whose economic resources are based on agricultural, agroindustrial, extractive, forestry and environmental conservation activities.
• Residential: Space destined almost exclusively for housing.
• Residential subsidized project (Residential Sub): Housing project subsidized by local Government.
• Industrial: Space destined to the industry, which is its wealth source.
• Official: Space that depends or comes directly from the State or from a recognized authority.
• Commercial: Commercial space or that is related to any commercial economic activity.
• Provisional: Temporary space, not definitive.
• Special: Adequate or exclusive space for a certain activity.
• Self-consumption: Space that can be self-sufficient with alternative energies.

Table 2 shows some instances of the power consumption-related features available in our data set. In addition, the number of instances for each location is provided in Table 3. Here, we include the number and percentage of instances of each municipality regarding the entire data set. As well, the latitude, longitude and entire population of each municipality is presented.

From Table 3, it can be observed that Policarpa amounts the highest percentage of instances (19.56%). As a whole, the data set is balanced by municipalities, excepting Magui and Roberto Payan that present fewer instances. Furthermore, Fig. 2 shows the range of values of power consumption per municipality between December 2010 and May 2016.

As it can be observed in Fig. 2, the consumption patterns are varied by municipality, but some regularities can be found by years. In this sense, Barbacoas municipality seems to have higher power consumption over all the years meanwhile lower consumptions belong to Cumbitara.

In Fig. 3, we include data distribution of the remaining four features available in the data set.

In Fig. 3, it can be noticed that there are more power consumption data in rural than urban sectors. Similarly, the use of power consumption of Residential is the one that presents the most instances while the least is for the industrial case. Regarding the year feature, the lowest number of instances is presented in 2010 and 2016, since for these cases no information was collected for the 12 months. Finally, the data is formed mostly (2138 cases) by low strata (=0) since the instances of this case represent 48.29% of the total data.

As a summary of the PCSTCOL data set, Table 4 shows a brief description of data types and range of values for each feature available in the data set.

In Table 4, a comprehensive description of features of the data set is observed. Three different data types are available. With four categorical, a character and numeric variable. Notice that categorical features might be discretized if necessary.

The R/R Studio software were used to perform the pre-processing and filtering procedures as well as to generate the statistical graphics. The software was run using a standard computer (Intel (R) Core (TM) i7-6500U, CPU @2.50 GHz, 8 GB RAM). The source code can be download from https://doi.org/10.17632/xbt7scz5ny.3.

## Footnotes

2 https://www.cedenar.com.co/webcedenar/resolucion-038-del-2014-codigo-de-medida/#1569851900090-6b331fa7-971e.
